# Entrepreneurial growth mindset boosts entrepreneurial intention via the serial path of cognitive flexibility and entrepreneurial alertness

**DOI:** 10.3389/fpsyg.2026.1901628

**Published:** 2026-07-24

**Authors:** Yaolishun Xing, Minjie Li, Xiaoming Jiang, Ying Qiao, Xinming Yu

**Affiliations:** 1 School of Innovation and Entrepreneurship, Wuchang Institute of Technology, Wuhan, Hubei, China; 2 School of Entrepreneurship, Xi'an International University, Xi'an, Shaanxi, China; 3 Science and Technology Division, Wuchang Institute of Technology, Wuhan, Hubei, China; 4 Academic Affairs Office, Hebei College of Science and Technology, Baoding, Hebei, China; 5 School of Intelligent Manufacturing, Wuchang Institute of Technology, Wuhan, Hubei, China

**Keywords:** cognitive flexibility, entrepreneurial alertness, entrepreneurial growth mindset, entrepreneurial intention, serial mediation, university students

## Abstract

This study investigates the association between entrepreneurial growth mindset and Chinese university students' entrepreneurial intention and its underlying psychological mechanisms, with a particular focus on the serial mediating roles of cognitive flexibility and entrepreneurial alertness. Using convenience sampling, 736 junior and senior students from three Chinese universities were recruited. Participants completed validated scales measuring entrepreneurial growth mindset, cognitive flexibility, entrepreneurial alertness, and entrepreneurial intention. Structural equation modeling was employed to test a serial mediation model, and indirect effects were evaluated using the bootstrap method with 5,000 resamples. Entrepreneurial growth mindset, cognitive flexibility, entrepreneurial alertness, and entrepreneurial intention were all positively and significantly correlated. Entrepreneurial growth mindset significantly and positively predicted entrepreneurial intention. Cognitive flexibility partially mediated the relationship between entrepreneurial growth mindset and entrepreneurial intention. Furthermore, cognitive flexibility and entrepreneurial alertness jointly constituted a significant serial mediation pathway. These findings are consistent with a multilevel cognitive mechanism through which entrepreneurial growth mindset influences entrepreneurial intention via cognitive flexibility and entrepreneurial alertness. The results offer a preliminary theoretical framework and practical roadmap for entrepreneurship education in higher education institutions.

## 1 Introduction

Entrepreneurship serves as a vital engine of economic development and offers university students diverse pathways for self-realization and professional growth. Against the backdrop of worldwide emphasis on innovation and entrepreneurship education, understanding how to effectively stimulate students' entrepreneurial intention has become a central theme in educational psychology and entrepreneurship research (Loi et al., 2023). However, despite sustained institutional investment in entrepreneurship curricula and incubation support systems, entrepreneurial intention among Chinese university students remains relatively subdued (Supriandi and Arisanti, 2025; Xing and Lee, 2025). According to the China College Graduate Employment Annual Report published by Mycos (2025), only 1.6% of Chinese undergraduate graduates chose self-employment in 2024, a figure substantially lower than that observed in many developed economies (Deng et al., 2024). This persistent gap suggests that external resources and coursework alone may be insufficient to fully mobilize students' entrepreneurial motivation. Consequently, there is an urgent need to examine the internal psychological and cognitive mechanisms that underlie the formation of entrepreneurial intention (Loi et al., 2023).

Entrepreneurial intention refers to the subjective probability that an individual plans to engage in entrepreneurial activity in the future and represents the most direct antecedent of actual entrepreneurial behavior (Ajzen, 1991). Extensive prior research has explained entrepreneurial intention from the perspectives of external environments-such as entrepreneurship education and social support-or general individual traits, including risk propensity and achievement motivation. Yet these approaches have paid insufficient attention to the deeper cognitive foundations that shape intention. Dweck's (2006) implicit theory provides a critical theoretical lens for addressing this gap. The theory distinguishes between two naive beliefs regarding the malleability of ability: entity theory, which holds that ability is fixed, and incremental theory, which holds that ability can be developed through effort and learning. Individuals who endorse an incremental theory-commonly referred to as a growth mindset-believe that competence can be continuously improved through deliberate practice, reflection, and learning (Jin et al., 2025). In recent years, this construct has been extended into the entrepreneurship domain, giving rise to the concept of entrepreneurial growth mindset. This construct is defined as an individual's incremental belief that entrepreneurial competence can be enhanced through deliberate practice, reflection, and continuous learning (Billingsley et al., 2021; Burnette et al., 2020). It is important to note that the general term entrepreneurial mindset typically denotes a holistic cognitive orientation encompassing value creation, opportunity recognition, and risk taking, whereas entrepreneurial growth mindset specifically concerns the metacognitive belief regarding whether entrepreneurial ability is malleable. As such, it represents a domain-specific extension of implicit theory into entrepreneurship (Billingsley et al., 2021; Dewi et al., 2025).

According to implicit theory, incremental theorists tend to adopt a mastery orientation, focusing on skill development rather than outcome evaluation when confronting challenges (Dweck, 2006). Therefore, individuals who hold an entrepreneurial growth mindset generally interpret failures as learning opportunities and are more likely to form positive behavioral intentions when facing uncertain entrepreneurial tasks (Billingsley et al., 2021; Burnette et al., 2020; Zhang G. et al., 2022). The positive association between entrepreneurial growth mindset and entrepreneurial intention rests on several logical foundations. First, an entrepreneurial growth mindset motivates individuals to actively engage in skill-building exercises, thereby enhancing their perceived feasibility of entrepreneurship and strengthening subjective willingness (Supriandi and Arisanti, 2025). Second, it attenuates psychological fear of failure and dismantles the psychological barriers that impede entrepreneurial decision-making (Zhang G. et al., 2022). Third, individuals with an entrepreneurial growth mindset are more inclined to proactively search for and exploit entrepreneurial opportunities, thereby generating novel business ideas (Botha, 2024). Empirical studies have consistently confirmed that entrepreneurial growth mindset significantly enhances entrepreneurial intention (Burnette et al., 2020; Dewi et al., 2025; Zhang G. et al., 2022). On the basis of this evidence, we propose Hypothesis 1: Entrepreneurial growth mindset has a significant positive effect on university students' entrepreneurial intention.

Beyond its direct influence, entrepreneurial growth mindset may also exert indirect effects through specific cognitive processes. Implicit theory not only shapes behavioral motivation but also influences the selection of cognitive strategies under uncertainty. Cognitive flexibility is a particularly important cognitive strategy in this regard. It refers to the capacity to break rigid mental frameworks, tolerate ambiguity, and consider multiple solutions to a given problem (Martin and Rubin, 1995). In the entrepreneurial context, cognitive flexibility manifests as the tendency to overcome path dependence, tolerate uncertainty, and entertain multiple options in parallel (Dheer and Lenartowicz, 2019; Wang et al., 2021). Research across diverse domains-including language learning and creative thinking-has demonstrated that a growth mindset significantly predicts cognitive flexibility (Jia et al., 2025; Zhou, 2024). A growth mindset stimulates the desire to explore new knowledge, and this cognitive openness directly enhances flexibility, enabling individuals to examine potential business opportunities from multiple perspectives (Heinemann et al., 2022).

Although the positive relationship between general growth mindset and cognitive flexibility has been established, direct empirical verification in the entrepreneurship context remains scarce. According to implicit theory, the mastery orientation driven by incremental beliefs prompts individuals to actively deploy more flexible cognitive strategies. This enhanced flexibility, in turn, facilitates entrepreneurial intention by reducing perceived cognitive costs in uncertain decision-making, strengthening self-efficacy beliefs regarding entrepreneurial challenges, and enabling the recognition of non-obvious business opportunities through flexible problem-solving (Bandura, 1997; Baron, 2006; Laureiro-Martínez et al., 2015). Thus, cognitive flexibility may serve as a critical mediating mechanism that transmits the influence of entrepreneurial growth mindset to entrepreneurial intention. Moreover, a substantial body of literature has documented that cognitive flexibility exerts a significant positive influence on entrepreneurial intention (Dheer and Lenartowicz, 2019; Mishra and Singh, 2022; Zhao et al., 2022). Therefore, we propose Hypothesis 2: Cognitive flexibility mediates the relationship between entrepreneurial growth mindset and entrepreneurial intention.

Entrepreneurial alertness denotes the cognitive propensity to identify, evaluate, and exploit entrepreneurial opportunities (Kirzner, 1973). Tang et al. (2012) decomposed this construct into three sub-dimensions: scanning and search, association and connection, and evaluation and judgment. Highly alert individuals actively scan the information environment, connect disparate information fragments, and assess the value of potential opportunities, thereby capturing business prospects that others may overlook (Araujo et al., 2023; Tang et al., 2012). Existing research indicates that cognitive flexibility substantially enhances entrepreneurial alertness (Gill et al., 2021; Wang et al., 2021; Yu et al., 2023; Zhang M. et al., 2022), while entrepreneurial alertness, in turn, positively influences entrepreneurial intention (Araujo et al., 2023; Hoang et al., 2023; Zhang M. et al., 2022). These findings suggest that entrepreneurial growth mindset may be linked to entrepreneurial intention through a serial pathway involving cognitive flexibility and entrepreneurial alertness.

Although a direct path from entrepreneurial growth mindset to entrepreneurial alertness may appear theoretically plausible, existing empirical evidence suggests a more complex relationship. Li et al. (2022) found that growth mindset only predicted entrepreneurial action under high challenge conditions, with its effect disappearing when situational demands were low. Billingsley et al. (2021) further observed that entrepreneurial growth mindset was negatively correlated with risk-taking, suggesting that a general belief in ability malleability may not straightforwardly translate into the exploratory behaviors characteristic of entrepreneurial alertness. Moreover, research on entrepreneurial alertness has consistently positioned it as a mediating mechanism driven by more specific cognitive and personality antecedents—such as creativity, proactive personality, and cognitive curiosity—rather than as a direct outcome of broad motivational beliefs (Hu et al., 2018; Heinemann et al., 2022). The logic of cognitive appraisal theory (Lazarus and Folkman, 1984) offers a useful lens for interpreting why the influence of entrepreneurial growth mindset on entrepreneurial alertness may be indirect. This logic suggests that when individuals encounter uncertain tasks, they proceed through two sequential stages: primary appraisal, in which they evaluate whether an event is threatening or challenging, and secondary appraisal, in which they mobilize specific cognitive strategies to cope with the situation. Prior research has shown that challenge appraisals are associated with greater cognitive flexibility and exploratory behavior, whereas threat appraisals tend to narrow attentional focus and reduce cognitive resources for problem-solving (Blascovich, 2008; Seery, 2011).

Applied to the present context, this logic suggests that individuals who hold an entrepreneurial growth mindset tend to appraise entrepreneurial uncertainty as a challenge rather than a threat during the primary appraisal stage. This challenge orientation then prompts them, during secondary appraisal, to deploy a broader repertoire of cognitive strategies, thereby increasing cognitive flexibility. As a crucial metacognitive capacity, cognitive flexibility enables individuals to transcend fixed mental schemas, tolerate ambiguity, and examine problems from multiple angles (Dajani and Uddin, 2015). Only within this flexible cognitive framework can individuals effectively direct attention toward the external environment, engage in information scanning, make cross-domain connections, and judge the value of opportunities—the very behaviors that constitute entrepreneurial alertness. In other words, entrepreneurial alertness is an executive-level tendency for opportunity recognition that requires cognitive flexibility as a prerequisite. Without a flexible cognitive architecture, a growth mindset remains at a general motivational level and may not automatically translate into systematic opportunity recognition behaviors. Consequently, the influence of entrepreneurial growth mindset on entrepreneurial alertness may be transmitted through cognitive flexibility rather than acting directly. Accordingly, we propose Hypothesis 3: Cognitive flexibility and entrepreneurial alertness serially mediate the relationship between entrepreneurial growth mindset and entrepreneurial intention.

In summary, the present study draws upon implicit theory as its primary theoretical foundation and uses the logic of cognitive appraisal theory as a conceptual lens for interpreting the hypothesized sequential relationships among the constructs. We examine the sequential transmission of cognitive flexibility and entrepreneurial alertness between entrepreneurial growth mindset and entrepreneurial intention, with the aim of uncovering the deep cognitive mechanisms underlying entrepreneurial intention formation and providing an actionable theoretical framework for entrepreneurship education.

## 2 Materials and methods

### 2.1 Participants

The study employed convenience sampling to recruit participants from three universities located in central, northern, and western China between March and April, 2026. These institutions were situated in regions with differing levels of economic development, which helped enhance the geographic heterogeneity of the sample and the external validity of the findings. In addition, the sampled universities boast robust innovation and entrepreneurship education resources. Among them, one institution has been accredited by the Ministry of Education as a national model university for innovation and entrepreneurship education, alongside two provincial-level demonstration centers for innovation and entrepreneurship. This sampling approach guarantees that all participants were recruited from universities with well-established, systematic innovation and entrepreneurship education systems, thereby enhancing the relevance and practical value of the research results for scholarship on entrepreneurship education in higher education. Junior and senior students were targeted because they face imminent career decisions between employment and entrepreneurship, have typically completed entrepreneurship education courses, and display heightened attention to entrepreneurial pathways.

Data were collected via an online survey platform (Wenjuanxing). Instructors who taught entrepreneurship courses at the three universities administered the questionnaire during class sessions. To enhance data quality, several procedural design features were implemented (Podsakoff et al., 2024): the survey was conducted anonymously to encourage honest responding; different scales were presented with varying response formats and instructions to reduce response inertia; attention check items were embedded to identify careless responses; and reverse-coded items were included in selected scales to minimize acquiescence bias. A total of 859 questionnaires were returned. Following standard data quality control procedures, we excluded responses that failed an embedded attention check item. The final sample comprised 736 valid responses, yielding an effective response rate of 85.68%.

The demographic composition of the sample is summarized in Table 1. Of the participants, 313 (42.53%) were male and 423 (57.47%) were female. In terms of academic year, 361 (49.05%) were juniors and 375 (50.95%) were seniors. With respect to major, 300 (40.76%) were enrolled in natural sciences and 436 (59.24%) in humanities and social sciences. A total of 390 participants (52.99%) reported deep involvement in entrepreneurial practice, whereas 346 (47.01%) did not. Deep involvement was operationally defined as having served as a core member in an entrepreneurial project for more than 3 months during university, or having won a prize at or above the university level in an entrepreneurship competition.

**Table 1:** Sample demographic characteristics.

Variable	Category	*N*	Percentage (%)
Gender	Male	313	42.53%
	Female	423	57.47%
Grade	Junior	361	49.05%
	Senior	375	50.95%
Major	Natural sciences	300	40.76%
	Humanities and social sciences	436	59.24%
Deep entrepreneurial practice	Yes	390	52.99%
	No	346	47.01%

### 2.2 Measures

All scales used in this study were well-established instruments. Responses were recorded on a 5-point Likert scale, with specific anchors varying by scale. One attention check item was embedded in the questionnaire to identify careless or random responding.

#### 2.2.1 Entrepreneurial growth mindset scale

Entrepreneurial Growth Mindset Scale adopted the three-item scale developed by Burnette et al. (2020) on the basis of Dweck's (2006) implicit theory. Items were rated on a 5-point scale ranging from 1 (strongly disagree) to 5 (strongly agree). All items were reverse-scored. A representative item reads: “You have a certain amount of entrepreneurial ability, and you can't really do much to change it.” In this study, Cronbach's alpha for the scale was 0.815, with higher scores indicating a stronger entrepreneurial growth mindset.

#### 2.2.2 Cognitive flexibility scale

Cognitive flexibility was measured using the eight-item scale adapted by Zhao et al. (2024) from the instrument originally developed by Martin and Rubin (1995). Items were rated on a 5-point scale from 1 (strongly disagree) to 5 (strongly agree). A sample item reads: “I can communicate an idea in many different ways.” Cronbach's alpha in the present study was 0.910.

#### 2.2.3 Entrepreneurial alertness scale

Entrepreneurial alertness was assessed with the 11-item revised version developed by Boso et al. (2019), based on the original 13-item scale by Tang et al. (2012). The scale encompasses three dimensions: scanning and search (e.g., “I always keep an eye out for new business ideas when looking for information.”), association and connection (e.g., “I see links between seemingly unrelated pieces of information.”), and evaluation and judgment (e.g., “I have a gut feeling for potential opportunities”). These three dimensions collectively reflect entrepreneurial alertness as a second-order latent variable. To validate this second-order structure, the study conducted a separate confirmatory factor analysis (CFA) on the entrepreneurial alertness scale. The results showed good model fit with χ^2^/df = 2.688, CFI = 0.982, TLI = 0.975, RMSEA = 0.048, SRMR = 0.031. This second-order factor structure was specified based on theoretical grounds (Tang et al., 2012; Boso et al., 2019) and determined prior to data analysis. Items were rated on a 5-point scale from 1 (strongly disagree) to 5 (strongly agree). The overall Cronbach's alpha in this study was 0.890.

#### 2.2.4 Entrepreneurial intention scale

Entrepreneurial intention was measured with the six-item scale developed by Liñán and Chen (2009). Items were rated on a 5-point scale from 1 (strongly disagree) to 5 (strongly agree). A sample item reads: “I am determined to create a firm in the future.” Cronbach's alpha for this scale in the present study was 0.915.

### 2.3 Data analysis

Data analysis was conducted using SPSS 27.0 and AMOS 26.0. Entrepreneurial alertness was specified as a second-order reflective construct, with its three first-order dimensions serving as indicators. The analytical procedure comprised the following steps:

- Common method bias assessment: We performed Harman's single-factor test by submitting all measurement items to an unrotated exploratory factor analysis to evaluate the severity of common method variance (Podsakoff et al., 2003).
- Reliability and validity assessment: Internal consistency was examined via Cronbach's alpha and composite reliability (CR). Convergent validity was assessed using the average variance extracted (AVE). Confirmatory factor analysis (CFA) was conducted in AMOS to evaluate structural validity and model fit.
- Descriptive statistics and correlation analysis: Means, standard deviations, and Pearson correlation coefficients were computed for all variables to provide a preliminary examination of bivariate relationships.
- Hypothesis testing: A structural equation model was constructed in AMOS for path analysis and mediation testing. Indirect effects were estimated using the bootstrap method with 5,000 resamples to generate bias-corrected 95% confidence intervals.
- Supplementary analysis: A series of hierarchical regression analyses with control variables (gender, grade, major, and deep entrepreneurial practice) was conducted in SPSS to examine the robustness of the main SEM findings.

## 3 Results

### 3.1 Common method bias assessment

Because all variables in this study were measured via self-report, common method bias could pose a threat to validity. We therefore conducted Harman's single-factor test on all scale items (Podsakoff et al., 2003). First, using SPSS 26, we performed an exploratory factor analysis with all items entered simultaneously and extracted six factors with eigenvalues greater than 1. The largest factor accounted for 32.75% of the total variance, which falls below the conventional 40% threshold (Zhang G. et al., 2022). Second, we conducted a full collinearity assessment following Kock's (2015) recommended procedure. We computed variance inflation factors (VIFs) for all constructs in the model, with each construct regressed on the other constructs. The VIF values ranged from 1.076 to 1.481, all well below the conservative threshold of 3.3 (Kock, 2015). Together, these results indicate that common method bias was not a serious concern in the present study.

### 3.2 Reliability and validity assessment

Because entrepreneurial alertness was modeled as a second-order latent variable, this study examined the reliability and validity of its three first-order dimensions along with the other latent variables. As shown in Table 2, Cronbach's alpha values for all latent variables exceeded 0.80, CR values exceeded 0.70, and AVE values exceeded 0.50. These results demonstrate satisfactory internal consistency and convergent validity. CFA further revealed that all standardized factor loadings were greater than 0.60 and significant at the *p* < 0.001 level.

**Table 2:** Reliability and convergent validity of core study variables.

Variable	α	CR	AVE
EGM	0.815	0.815	0.595
CF	0.910	0.910	0.560
SS (Scanning and Search)	0.841	0.843	0.574
AC (Association and Connection)	0.774	0.789	0.557
EJ (Evaluation and Judgment)	0.860	0.861	0.607
EI	0.915	0.916	0.647

EGM, entrepreneurial growth mindset; CF, cognitive flexibility; EI, entrepreneurial intention; EA (entrepreneurial alertness) is the second-order construct formed by SS, AC, EJ.

To further examine discriminant validity, a series of confirmatory factor were analyzed in AMOS 26.0 and compared the hypothesized measurement model against alternative measurement models (including one-factor and multi-factor models) to assess discriminant validity. As shown in Table 3, the six-factor model provided the best fit to the data: χ^2^/df = 1.955, CFI = 0.971, TLI = 0.968, RMSEA = 0.036, SRMR = 0.033. All factor loadings exceeded 0.50 and were significant at *p* < 0.001, indicating that the items adequately represented their respective latent constructs (Wu, 2009). These results confirm that the measurement model fit the data well.

**Table 3:** Model fit comparisons across competing confirmatory factor structures.

Model	χ^2^	df	χ^2^/df	CFI	TLI	RMSEA	SRMR
Six-factor	654.798	335	1.955	0.971	0.968	0.036	0.033
Five-factor	974.051	340	2.865	0.943	0.937	0.062	0.050
Four-factor	1,807.497	345	5.239	0.869	0.856	0.076	0.099
Three-factor	4,150.005	348	11.925	0.659	0.630	0.122	0.134
Two-factor	5,110.454	349	14.643	0.573	0.538	0.136	0.125
Single-factor	5,775.558	350	16.502	0.513	0.475	0.145	0.132

Six-factor model: EGM, CF, scanning-search, association-connection, evaluation-judgment, EI.

The CFA results confirmed that the six-factor model fit the data significantly better than any competing model, indicating adequate discriminant validity among the constructs. Given that entrepreneurial alertness was conceptualized as comprising scanning and search, association and connection, and evaluation and judgment, the study additionally tested a second-order model for entrepreneurial alertness. The second-order model exhibited good fit: χ^2^/df = 2.045, CFI = 0.978, TLI = 0.975, RMSEA = 0.038, SRMR = 0.031. Consequently, entrepreneurial alertness was treated as a second-order latent variable in all subsequent analyses.

### 3.3 Descriptive statistics and correlation analysis

Table 4 presents the means, standard deviations, and Pearson correlation coefficients for all variables. The mean scores ranged from 3.161 to 3.412, indicating a moderate-to-high overall level across the constructs. Entrepreneurial growth mindset was positively and significantly correlated with cognitive flexibility (*r* = 0.266, *p* < 0.001), entrepreneurial intention (*r* = 0.305, *p* < 0.001), and entrepreneurial alertness (*r* = 0.154, *p* < 0.001). Cognitive flexibility was positively and significantly correlated with entrepreneurial alertness (*r* = 0.539, *p* < 0.001) and entrepreneurial intention (*r* = 0.355, *p* < 0.001). Entrepreneurial alertness was also positively and significantly correlated with entrepreneurial intention (*r* = 0.316, *p* < 0.001). These patterns provide preliminary support for the hypothesized relationships.

**Table 4:** Descriptive statistics and zero-order correlations among core constructs.

Variable	M	SD	EGM	CF	EA	EI
EGM	3.161	0.695	1			
CF	3.412	0.556	0.266^***^	1		
EA	3.350	0.475	0.154^***^	0.539^***^	1	
EI	3.175	0.606	0.305^***^	0.355^***^	0.316^***^	1

^***^p < 0.001.

### 3.4 Hypothesis testing

#### 3.4.1 Direct effect testing

**Figure 1:**
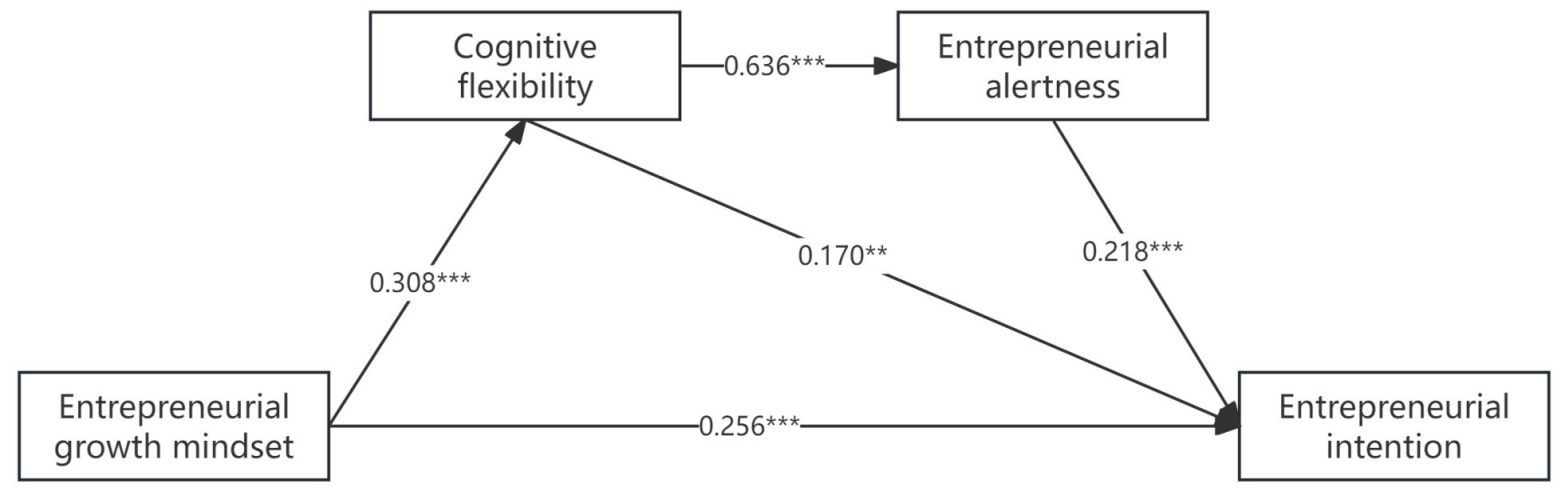
Serial mediation model with standardized path coefficients ^**^p < 0.01, ^***^p < 0.001.

The hypothesized paths were tested via structural equation modeling. The model demonstrated good fit: χ^2^/df = 2.032, CFI = 0.978, TLI = 0.975, RMSEA = 0.037, SRMR = 0.031. The model explained 9.5% of the variance in cognitive flexibility, 40.5% of the variance in entrepreneurial alertness, and 23.8% of the variance in entrepreneurial intention. As shown in Figure 1, entrepreneurial growth mindset had a significant direct effect on entrepreneurial intention (β = 0.256, *p* < 0.001), thereby supporting Hypothesis 1. In addition, entrepreneurial growth mindset significantly and positively predicted cognitive flexibility (β = 0.308, *p* < 0.001). Cognitive flexibility, in turn, significantly and positively predicted entrepreneurial alertness (β = 0.636, *p* < 0.001) and entrepreneurial intention (β = 0.170, *p* < 0.01). Entrepreneurial alertness also exerted a significant positive effect on entrepreneurial intention (β = 0.218, *p* < 0.001). The standardized path coefficients are detailed in Table 5. Consistent with Cohen (1988) criteria, the standardized path coefficients in this model range from 0.170 to 0.636. The coefficient capturing the predictive effect of cognitive flexibility on entrepreneurial alertness indicated a large effect (β = 0.636), and the remaining paths reflected small-to-medium effects.

**Table 5:** Standardized and unstandardized structural path coefficients.

Predictor → Outcome	B	SE	β	C.R.
EGM → CF	0.260	0.037	0.308	7.013^***^
CF → EA	0.507	0.039	0.636	12.964^***^
CF → EI	0.193	0.062	0.170	3.130^**^
EA → EI	0.310	0.080	0.218	3.865^***^
EGM → EI	0.245	0.040	0.256	6.090^***^

^**^p < 0.01, ^***^p < 0.001.

#### 3.4.2 Indirect effect testing

Bootstrap mediation analysis with 5,000 resamples was conducted via AMOS. The results are summarized in Table 6. The indirect path from entrepreneurial growth mindset to entrepreneurial intention via cognitive flexibility alone yielded an effect of 0.052, with a 95% percentile confidence interval of [0.008, 0.112]. Because this interval did not contain zero, the specific mediation effect of cognitive flexibility was significant, supporting Hypothesis 2. The serial indirect path from entrepreneurial growth mindset to entrepreneurial intention via cognitive flexibility and entrepreneurial alertness yielded an effect of 0.043, with a 95% confidence interval of [0.010, 0.085]. This interval also excluded zero, indicating that the serial mediation effect of cognitive flexibility and entrepreneurial alertness was significant, thereby supporting Hypothesis 3.

**Table 6:** Bootstrap indirect effect decomposition for mediation pathways.

Indirect path	Effect size	Boot SE	95% percentile CI (lower)	95% percentile CI (upper)	Proportion of total effect (%)
EGM → CF → EI	0.052	0.026	0.008	0.112	14.82%
EGM → CF → EA → EI	0.043	0.019	0.010	0.085	12.25%
Total indirect effect	0.095	0.024	0.053	0.146	27.07%
Total effect	0.351	0.046	0.257	0.439	–

In summary, all hypotheses proposed in this study were supported by the data. Entrepreneurial growth mindset demonstrated a significant direct positive effect on entrepreneurial intention (Hypothesis 1 supported). Cognitive flexibility played a significant mediating role (Hypothesis 2 supported). Finally, cognitive flexibility and entrepreneurial alertness jointly formed a complete serial mediation mechanism (Hypothesis 3 supported).

### 3.5 Regression analysis with control variables

To examine whether the observed relationships remained robust after accounting for potential confounding demographic characteristics, the study conducted a series of regression analyses with control variables. Demographic variables (gender, grade, major, and deep entrepreneurial practice) were entered in Step 1, followed by the core predictors (entrepreneurial growth mindset, cognitive flexibility, and entrepreneurial alertness) in Step 2. Separate regressions were performed for each endogenous variable.

As shown in Table 7, for entrepreneurial intention, the final model explained 21.2% of the variance (R^2^ = 0.212, *F* (7, 728) = 27.973, *p* < 0.001), with a significant R^2^ change of 0.182 (*p* < 0.001) after adding the core predictors. All three core predictors remained statistically significant: entrepreneurial growth mindset (β = 0.221, *p* < 0.001), cognitive flexibility (β = 0.211, *p* < 0.001), and entrepreneurial alertness (β = 0.152, *p* < 0.001). Among the control variables, deep entrepreneurial practice (coded as 1 = with experience, 2 = without experience) showed a significant negative association with entrepreneurial intention (β = −0.104, *p* < 0.01), indicating that students with prior hands-on experience reported higher intention than those without. No other demographic variables were significant predictors (all ps > 0.05).

**Table 7:** Regression results with control variables.

Variable	CF	EA	EI
Gender	0.004	−0.042	−0.054
Grade	−0.055	−0.012	0.059
Major	−0.087^*^	0.053	0.031
Deep entrepreneurial practice	−0.099^**^	−0.119^***^	−0.104^**^
EGM	0.268^***^	0.015	0.221^***^
CF	—	0.527^***^	0.211^***^
EA	—	—	0.152^***^
*R* ^2^	0.098	0.307	0.212
Δ*R*^2^	0.071^***^	0.275^***^	0.182^***^
*F*	15.803^***^	53.850^***^	27.973^***^

Entries are standardized regression coefficients; ^*^p < 0.05, ^**^p < 0.01, ^***^p < 0.001.

The regression results for cognitive flexibility and entrepreneurial alertness were consistent with the SEM findings (see Table 7 for full results). Together, these analyses confirm that the predictive effects of the core psychological constructs on entrepreneurial intention remain statistically significant after controlling for demographic characteristics.

## 4 Discussion

### 4.1 Theoretical implications

The findings of this study offer several insights into how entrepreneurial growth mindset relates to entrepreneurial intention among Chinese university students. First, by examining entrepreneurial growth mindset, cognitive flexibility, entrepreneurial alertness, and entrepreneurial intention within a single serial mediation framework, the study extends prior research that has largely examined these relationships in isolation. Second, by identifying cognitive flexibility as a mediator between growth mindset and entrepreneurial outcomes in the entrepreneurship domain, the study provides empirical support for a mechanism previously theorized but largely untested in this context. Third, by revealing the hierarchical structure of entrepreneurial cognition—from foundational beliefs to cognitive strategies to executive-level tendencies—the study offers a more nuanced account of how motivational beliefs translate into entrepreneurial intentions. These contributions collectively provide a foundation for future research on the cognitive mechanisms underlying entrepreneurial intention formation.

#### 4.1.1 Direct effect of entrepreneurial growth mindset on entrepreneurial intention

The present study provides evidence that entrepreneurial growth mindset is positively associated with university students' entrepreneurial intention, a finding that aligns with prior empirical work (Burnette et al., 2020; Dewi et al., 2025; Lee et al., 2020). This result supports the applicability of implicit theory in the entrepreneurship context and demonstrates that individuals who believe entrepreneurial competence is malleable are more likely to develop entrepreneurial intentions. An entrepreneurial growth mindset may lead individuals to interpret entrepreneurial challenges as opportunities for skill enhancement rather than as tests of fixed ability, which is in turn associated with greater willingness to practice skills, reducing fear of failure, and actively searching for entrepreneurial opportunities (Botha, 2024; Supriandi and Arisanti, 2025; Zhang G. et al., 2022). Unlike much of the previous literature, which has focused predominantly on external environmental factors such as entrepreneurship education and social support, the present study highlights the independent contribution of internal belief systems. This emphasis on intrapersonal cognitive foundations complements existing extrinsic models and underscores the necessity of integrating psychological constructs into entrepreneurship research.

The persistently low entrepreneurial intention among Chinese university students—evidenced by the fact that only 1.6% of graduates choose self-employment—cannot be fully explained by resource scarcity or curriculum deficiencies alone. Recent evidence suggests that even when universities provide substantial entrepreneurial support, the effect on students' intentions remains weak unless students possess the internal capacity to translate such support into actionable motivation (Tseng, 2023). The findings suggest that entrepreneurial growth mindset may function as an internal engine that helps translate external educational inputs into entrepreneurial readiness. Without this foundational belief in the malleability of entrepreneurial competence, students may passively receive information without forming the proactive intention necessary to initiate venture creation. This insight is particularly relevant for the Chinese context, where cultural emphasis on academic credentialism and risk aversion may reinforce fixed beliefs about occupational identity, thereby suppressing entrepreneurial experimentation among university students. Moreover, the direct effect of entrepreneurial growth mindset on entrepreneurial intention remained the dominant pathway in our model, indicating that entrepreneurship education should prioritize the cultivation of students‘ growth mindset beliefs as a foundational objective, with supplementary cognitive training designed to enhance cognitive flexibility and entrepreneurial alertness.

#### 4.1.2 Independent mediating role of cognitive flexibility

This study provides evidence supporting cognitive flexibility as an independent mediator between entrepreneurial growth mindset and entrepreneurial intention, thereby extending prior research on the positive association between general growth mindset and cognitive flexibility into the entrepreneurship context (Zhou, 2024; Jia et al., 2025). From the perspective of implicit theory, entrepreneurial growth mindset is essentially a metacognitive belief about the malleability of entrepreneurial competence. Individuals who endorse this incremental belief tend to adopt a mastery orientation when confronting challenges, directing their attention toward skill development rather than outcome evaluation (Burnette et al., 2020; Billingsley et al., 2021). This mastery orientation generates intrinsic motivation to explore diverse solutions and fosters cognitive openness and curiosity—psychological tendencies that lie at the core of cognitive flexibility (Heinemann et al., 2022).

The logic of cognitive appraisal theory offers a useful framework for interpreting this transformation (Lazarus and Folkman, 1984). Within this framework, an entrepreneurial growth mindset may lead to evaluate entrepreneurial uncertainty as a challenge rather than a threat during primary appraisal, thereby reducing psychological defensiveness. During secondary appraisal, these individuals are more likely to mobilize a diversified repertoire of cognitive strategies, actively breaking mental set, tolerating ambiguity, and examining problems from multiple perspectives. Empirical evidence has further shown that cognitive flexibility enhances individuals' confidence in overcoming entrepreneurial obstacles and strengthens their perceived feasibility of entrepreneurship (Dheer and Lenartowicz, 2019; Wang et al., 2021; Mishra and Singh, 2022), thereby facilitating the formation of entrepreneurial intention. The logic of cognitive appraisal theory thus offers a useful framework for interpreting the transition from metacognitive belief to cognitive strategy and ultimately to behavioral intention.

Furthermore, the robust association of cognitive flexibility observed in the present model carries implications for understanding why some students may benefit from entrepreneurship education while others do not. Prior research has noted that entrepreneurship education often yields inconsistent effects on intention, with some studies reporting positive outcomes and others finding negligible or even negative effects (Liu et al., 2025). One plausible explanation for this inconsistency is that students vary in their baseline cognitive flexibility, which may influence whether they can effectively assimilate and apply the diverse, often contradictory information presented in entrepreneurship courses. Students high in cognitive flexibility can reconcile competing business models and adapt frameworks across contexts, whereas those low in flexibility may experience cognitive overload. Nevertheless, the mediating role of cognitive flexibility was partial rather than complete, implying that entrepreneurial growth mindset may influence entrepreneurial intention through additional mechanisms not captured in the current model.

#### 4.1.3 Serial mediating role of cognitive flexibility and entrepreneurial alertness

An important contribution of this study lies in the identification of a serial mediation pathway in which cognitive flexibility and entrepreneurial alertness are sequentially associated with the influence of entrepreneurial growth mindset to entrepreneurial intention. This pattern is interpretable through the lens of cognitive appraisal theory (Lazarus and Folkman, 1984): the challenge orientation formed during primary appraisal appears to be linked to concrete cognitive tendencies-namely, entrepreneurial alertness-through the cognitive flexibility deployed during secondary appraisal, which is in turn associated with entrepreneurial intention.

Our findings are consistent with the view that cognitive flexibility is associated with entrepreneurial alertness, in line with previous research conducted in different cultural and educational contexts (Wang et al., 2021; Gill et al., 2021). This implies that a general belief in the malleability of entrepreneurial ability may be insufficient, by itself, to elevate alertness levels. Alertness activities-such as scanning information, forming unconventional associations, and judging opportunity value-require more specific cognitive operations as underpinning. As Baron (2006) noted, only individuals who possess a flexible mental framework can effectively direct attention toward the external environment and recognize opportunities. The strong predictive effect of cognitive flexibility on entrepreneurial alertness observed in the present study further supports the theoretical position that cognitive flexibility plays a pivotal role in shaping individuals' attentiveness to entrepreneurial opportunities.

The serial mediation finding also advances our understanding of the hierarchical organization of entrepreneurial cognition. Rather than treating entrepreneurial alertness as an isolated trait that can be trained through simple exposure to business cases, our results suggest that alertness may be a higher-order cognitive output that is associated with the foundational capacity of cognitive flexibility. From an educational standpoint, this may suggest that interventions targeting alertness without first establishing cognitive flexibility may yield limited returns, as students lack the cognitive architecture necessary to sustain systematic opportunity scanning and cross-domain association.

Moreover, this study validates the positive association between entrepreneurial alertness and entrepreneurial intention, consistent with the original theoretical framework proposed by Tang et al. (2012) and subsequent meta-analytic conclusions (Araujo et al., 2023). By mapping the serial relationship between entrepreneurial growth mindset and entrepreneurial intention, the present study extends implicit theory from general ability beliefs into the entrepreneurship domain and resonates with the association between growth mindset and cognitive flexibility documented in language learning contexts (Zhou, 2024). The cross-situational consistency of these findings (Wang et al., 2021; Wei and Liu, 2020) indicates that entrepreneurial alertness often functions as an intermediate link within broader cognitive transmission chains and should be understood within a comprehensive cognitive framework rather than in isolation.

### 4.2 Practical implications

The findings of this study offer preliminary implications for entrepreneurship education. The direct effect of entrepreneurial growth mindset on entrepreneurial intention remained the dominant pathway in the present model, suggesting that entrepreneurship education could prioritize fostering students' growth mindset beliefs as a foundational objective, complemented by sequential training in cognitive flexibility and entrepreneurial alertness. The tripartite relationship identified here offers a staged configuration for such educational efforts.

To begin with, educators could consider cultivating an entrepreneurial growth mindset. This can be achieved by sharing entrepreneurial growth stories, designing low-threshold success experiences, and guiding students to reflect on failure as a learning opportunity. These practices help students internalize the belief that entrepreneurs are made rather than born. In practice, this may involve inviting alumni entrepreneurs to discuss their iterative learning trajectories, emphasizing how early venture failures contributed to subsequent success. Instructors should explicitly frame these narratives within an incremental framework, highlighting the role of deliberate practice and strategic adaptation rather than innate talent.

Building on this foundation, instructors may consider training cognitive flexibility. Dedicated modules targeting cognitive flexibility can be embedded within entrepreneurship courses. Specific exercises include multi-perspective case analyses in which students examine the same problem from the standpoints of different stakeholders, reverse brainstorming that challenges conventional assumptions, and scenario-based simulations requiring adaptive responses to unexpected disruptions. These activities help students break free from rigid thinking patterns. For instance, a design-thinking workshop might require students to reconceptualize a failing campus business from the perspectives of customers, investors, regulators, and community members within a compressed timeframe, thereby forcing rapid cognitive switching and perspective-taking.

Following the development of cognitive flexibility, curriculum could be designed to enhance entrepreneurial alertness. Students could be guided through deliberate practice in opportunity recognition and evaluation, such as scanning market information, making cross-domain connections, and assessing the feasibility of business ideas. These exercises translate flexible cognitive frameworks into concrete opportunity-identification capabilities. For example, instructors might assign students to monitor emerging trends across unrelated industries—such as biotechnology, social media, and sustainable agriculture—and then synthesize these observations into novel venture concepts. This practice mirrors the scanning-and-connection processes central to entrepreneurial alertness and ensures that cognitive flexibility is channeled toward actionable opportunity recognition rather than remaining an abstract intellectual capacity.

These three areas are interconnected and sequentially reinforcing, corresponding to the sequential pathway identified in the present model. Entrepreneurship education could follow an inward-to-outward progression, starting with the cultivation of foundational beliefs, then moving to cognitive strategies, and finally to executive-level tendencies, rather than isolating the transmission of knowledge or tools from the development of underlying cognitive capacities. Program evaluation could also consider whether students demonstrate measurable improvements in cognitive flexibility and alertness as intermediate outcomes, alongside changes in entrepreneurial intention.

### 4.3 Limitations and future directions

Several limitations of this study should be acknowledged. First, the cross-sectional design precludes strict causal inference. Although structural equation modeling and bootstrap techniques provide robust estimates of indirect effects under the assumption of theoretical directionality, the temporal ordering of variables cannot be definitively established with cross-sectional data. Future research may adopt longitudinal tracking designs or experimental manipulations to establish causality. A particularly promising approach would involve a pretest-posttest randomized controlled trial in which students receive a growth mindset intervention followed by cognitive flexibility training, with entrepreneurial intention and alertness measured at multiple follow-up points.

Second, entrepreneurial alertness was treated as a second-order construct in the present study, and its three sub-dimensions-scanning and search, association and connection, and evaluation and judgment-may play distinct roles along the serial pathway. Because the current analysis examined entrepreneurial alertness as a unified construct, the unique mediating contributions of each sub-dimension remain unexplored. Future studies should disaggregate these dimensions to test their differential effects. For example, it is possible that cognitive flexibility primarily facilitates the association and connection dimension by enabling cross-domain linkage, whereas the scanning and search dimension may depend more on motivational factors such as curiosity or intrinsic interest.

Third, the sample was drawn from three Chinese universities and was limited to junior and senior students. Caution is warranted when generalizing the findings to other cultural contexts or educational levels. Cross-cultural comparative research would be valuable for assessing the stability of the model across populations.

Fourth, while the present study used the logic of cognitive appraisal theory as a conceptual lens for interpreting the sequential relationships in the model, the primary and secondary appraisal processes were not directly measured. Future research could incorporate validated measures of cognitive appraisal to empirically test whether these processes operated as theorized.

Finally, all measures were self-reported, which may introduce shared method variance despite our statistical checks. Future studies could incorporate behavioral measures of cognitive flexibility—such as task-switching paradigms or the Wisconsin Card Sorting Test—and objective assessments of entrepreneurial alertness, such as opportunity recognition exercises with coded performance metrics. Additionally, physiological measures including resting-state functional magnetic resonance imaging could provide convergent evidence for the neural substrates of the cognitive flexibility-alertness link suggested by our theoretical model.

## 5 Conclusion

This study yields three main conclusions. First, entrepreneurial growth mindset is positively and significantly associated with entrepreneurial intention among university students, providing evidence for both a direct effect and indirect effects through cognitive flexibility and entrepreneurial alertness. Second, cognitive flexibility serves as an independent mediator in the relationship between entrepreneurial growth mindset and entrepreneurial intention. Third, cognitive flexibility and entrepreneurial alertness constitute a sequential serial mediation pathway: entrepreneurial growth mindset is positively associated with cognitive flexibility, which in turn is associated entrepreneurial alertness, and this sequential pathway is further linked to entrepreneurial intention. These findings are consistent with the proposed theoretical framework, which builds upon implicit theory and uses the sequential logic of cognitive appraisal theory as an interpretive lens for understanding the hierarchical relationships among the constructs. The results support the application of implicit theory in the entrepreneurship that moves from mindset cultivation to cognitive training and finally to alertness development.

## Data Availability

The original contributions presented in the study are included in the article/supplementary material, further inquiries can be directed to the corresponding author/s.
